# Microbe Producing Results Similar to the Ray Fungus

**Published:** 1896-11

**Authors:** 


PROFESSOR SAVTCHENKE (*La Presse Médicale*) has recently described a new microbe which produces a disease similar to that caused by the fungus of ordinary actinomycosis.—*Modern Medicine and Bacteriological Review*.

